# Stereotactic ablative radiotherapy for early-stage lung cancer following double lung transplantation

**DOI:** 10.1186/s13014-018-1089-8

**Published:** 2018-08-07

**Authors:** Hanbo Chen, Jussi Tikkanen, R. Gabriel Boldt, Alexander V. Louie

**Affiliations:** 1 Department of Radiation Oncology, London Health Sciences Centre, 790 Commissioners Road East, London, ON N6A 4L6 Canada; 2 Multi-Organ Transplant Program, Toronto General Hospital, University Health Network, 200 Elizabeth Street, Toronto, ON M5G 2C4 Canada

**Keywords:** Stereotactic body radiotherapy, Stereotactic ablative radiotherapy, Early stage, Non-small cell lung cancer, Lung transplantation

## Abstract

**Background:**

Development of primary lung cancer in donor lung post-lung transplantation is very rare, with few described cases. The safety of stereotactic ablative radiotherapy (SABR) for early-stage lung cancer arising from donor lung is unclear.

**Case presentation:**

Herein, we present a case of a patient with a Stage IB adenocarcinoma arising from donor lung 8 years post-double lung transplantation, which was performed due to advanced emphysema. The patient was ineligible for surgical management due to chronic lung allograft dysfunction, which significantly compromised pulmonary function. Full dose SABR was delivered with curative intent after a discussion with the patient. The patient tolerated the treatment well, with one episode of subacute toxicity that resolved with treatment. There was no evidence of recurrence at 15 months post-treatment and the patient’s pulmonary function did not deviate from the pre-SABR baseline.

**Conclusions:**

SABR appears feasible for medically-inoperable early-stage primary lung adenocarcinoma in the setting of previous double-lung transplantation.

## Background

The development of primary bronchogenic carcinoma following double-lung transplantation is very rare [1]. The use of stereotactic ablative radiotherapy (SABR) for medically-inoperable early-stage non-small cell lung cancer (NSCLC) arising from donor lung following double-lung transplantation has not been previously described. Here we present a case where SABR was used safely to treat an early-stage lung adenocarcinoma in a patient with a history of double-lung transplantation.

## Case presentation

Our patient is a 60-year-old female with severe emphysema who underwent a double-lung transplant in 2008, donated from a 64-year-old female with a 25-pack-year smoking history, stopping in 1982. The explanted lungs showed signs of severe emphysema but no malignant features. Post-transplant imaging demonstrated a mild to moderate degree of emphysema in the donor lungs. The patient received triple-drug immunosuppression with cyclosporine A, azathioprine, and prednisone and remained well until 2014, when she developed post-transplant lymphoproliferative disorder (PTLD), requiring chemotherapy with cyclophosphamide/doxorubicin/vincristine/prednisone-rituximab and discontinuation of azathioprine. While her PTLD is currently in remission, she subsequently developed chronic lung allograft dysfunction (CLAD) in 2015, with a marked decline in forced expiratory volume in 1 s (FEV1) from the post-transplant baseline of 3.1 L to 0.9 L. Her FEV1 has been stable at 0.9 L since 2015.

In 2016, the patient presented with a solitary right upper lobe pulmonary nodule on routine computed tomography (CT) scan. Following serial growth (12 mm) on CT, the lung nodule was biopsied, revealing primary lung adenocarcinoma. The pathologic sample was negative for both the ALK fusion oncogene and EGFR mutation. Staging whole-body positron-emission tomography-computerized tomography (PET-CT) scan and magnetic resonance (MR) scan of the brain did not reveal any evidence of distant metastases.

**Fig. 1:**
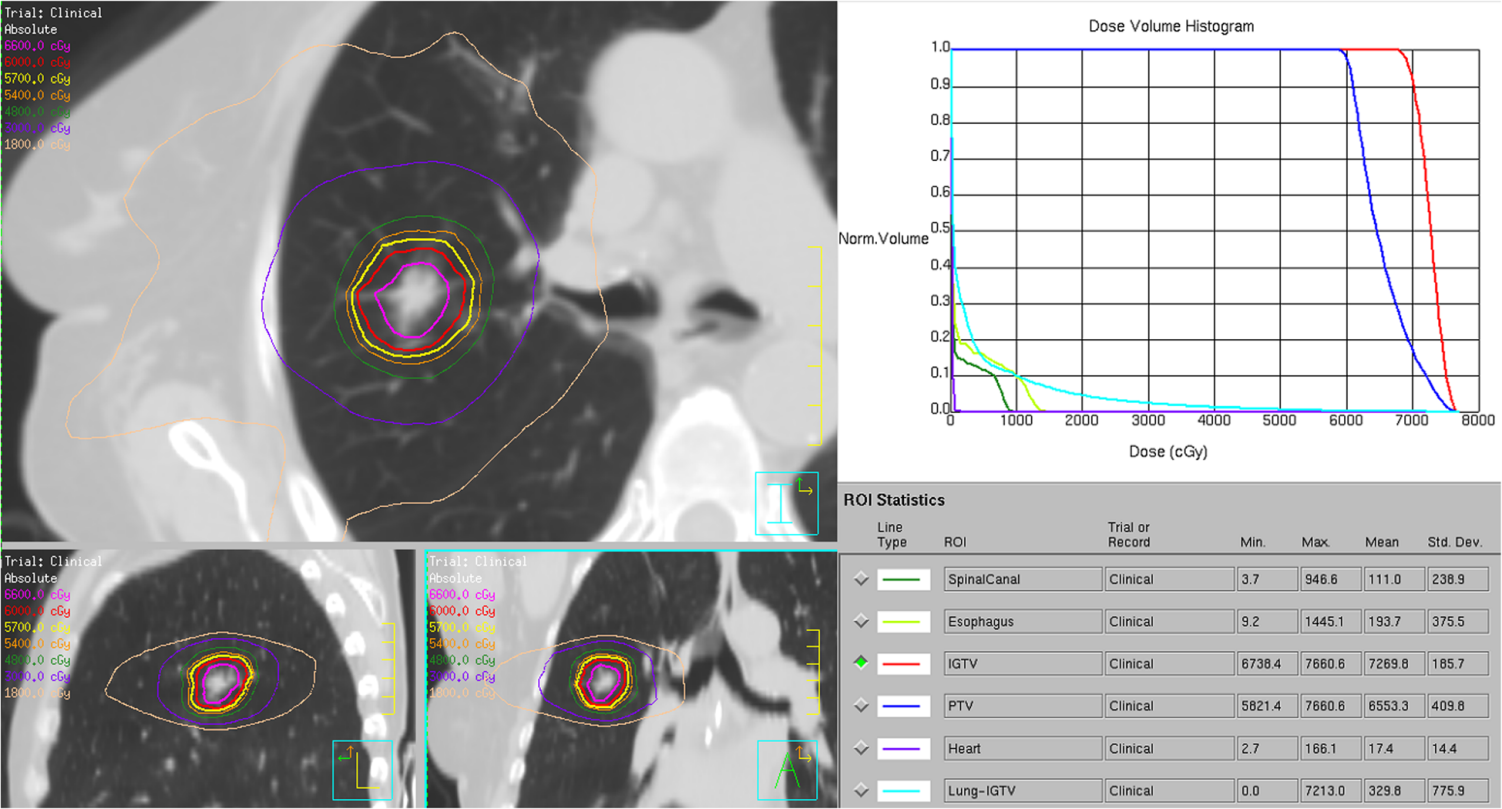
Radiotherapy plan demonstrating isodose distributions and the dose-volume histogram for the targets (internal gross tumor volume (IGTV) – the same as internal target volume (ITV) and planning target volume (PTV)) and organs-at-risk (spinal canal, esophagus, heart and lung)

The patient was not a surgical candidate due to her poor pulmonary function (FEV1 = 0.9 L, FEV1/forced vital capacity [FVC] = 39%). She consented to undergo SABR. 4D-CT simulation was performed with vacuum cushion immobilization and the gross tumour volume (GTV) was contoured on the end-inspiratory and end-expiratory phases. No margin for microscopic disease extension was used. An internal target volume (ITV) was generated by merging the end-inspiratory and end-expiratory GTVs and a margin of 0.5 cm around the ITV was used to generate the planning target volume (PTV). Free-breathing treatment delivery was chosen due to minimal tumour respiratory motion. A risk-adapted schedule of 60 Gy in 8 fractions (biologically effective dose = 105 Gy_10_) was delivered every other day via a flattening filter-free volumetric modulated arc therapy technique using two 225-degree 6MV arcs on a linear accelerator. The dose was prescribed to the 80% isodose line, which encompassed 97.5% of the planning target volume. Planning was performed on Pinnacle [2] version 9.10 with heterogeneity correction, using published dose constraints for normal tissues [3]. The mean lung dose was 3.3 Gy, and the volume of lung receiving ≥ 20 Gy or more (V20) was 4.3%. She completed radiotherapy in February 2017. Figure 1 shows selected images from the patient’s radiotherapy plan.

Two months following completion of SABR, the patient was admitted to a community hospital with dyspnea, hypoxia, tachypnea and increased production of yellow sputum. She was afebrile, with no leukocytosis. A CT pulmonary angiogram did not demonstrate pulmonary embolism, though bilateral inflammatory opacities were suspicious for an infectious etiology. There was no evidence of an inflammatory process geometrically associated with the radiotherapy field suspicious for radiation pneumonitis. Sputum cultures did not identify a causative organism, though the patient was started on antibiotics empirically prior to obtaining a sputum sample and a viral cause was also possible. After discussion with the Respirology and Radiation Oncology services, she was started on cefuroxime and prednisone 50 mg daily to cover all potential etiologies. Her respiratory function returned to baseline and she was discharged after 12 days of admission with a tapering schedule of prednisone. A follow-up CT 1 month later showed resolution of the bilateral opacities.

**Fig. 2:**
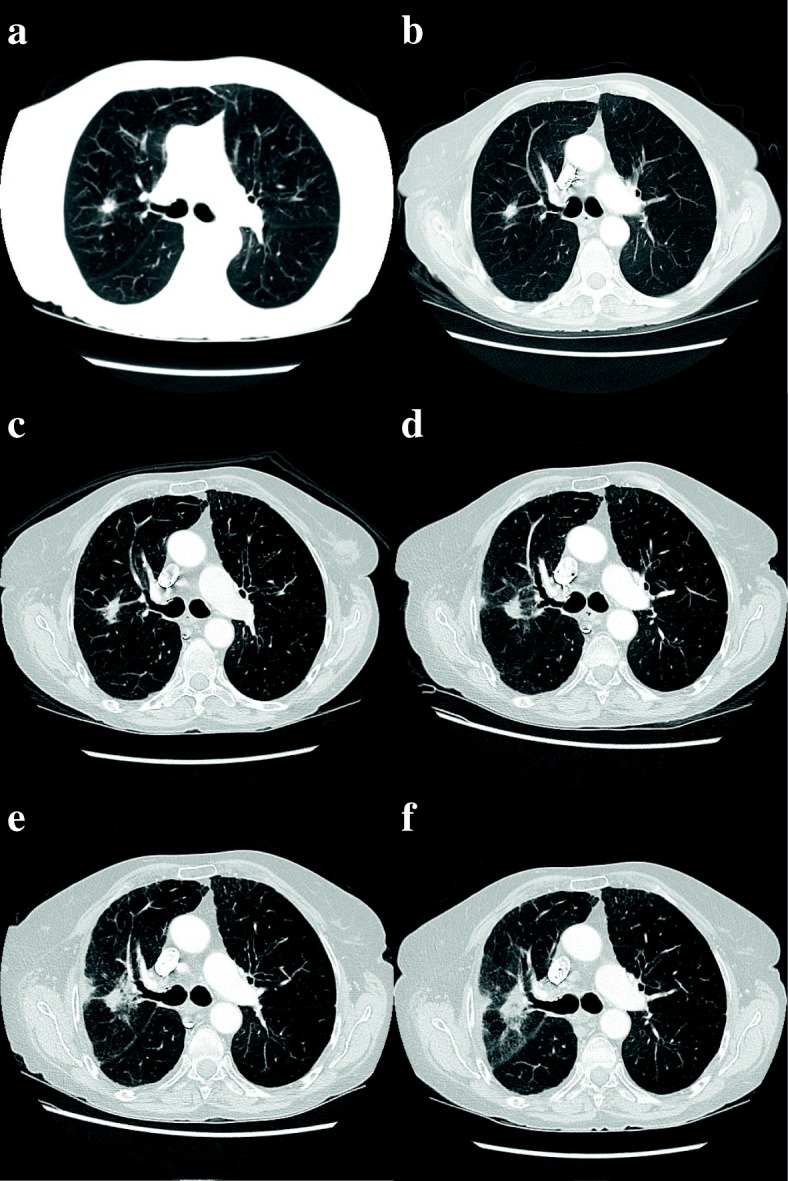
CT images demonstrating the primary lesion **a** pre-treatment, **b** 3 months, **c** 6 months, **d** 9 months and, **e** 12 months and **f** 15 months post-treatment. Evolving post-radiation changes are observed

Since discharge, our patient’s pulmonary function and subjective dyspnea have returned to pre-treatment levels and remained stable. Her most recent FEV1 was 0.9 L, with FEV1/FVC of 37% in March of 2018. Surveillance CTs at regular intervals demonstrated good local control, and post-treatment fibrotic pulmonary changes that are common following SABR (Fig. 2). To date, the radiographic fibrotic changes do not harbor adverse features [2] that would be suggestive of recurrence and/or warrant PET-CT scan or repeat biopsy. The patient is alive and well as of May 2018.

## Discussion and conclusions

Though patients are at higher risks of developing cancer post-solid organ transplantation [4], primary lung cancer developing in donor lung following lung transplantation is exceedingly rare. A recent review identified 14 patients among 11 sources between 2001 and 2012, with estimated prevalence ranging from 0.3 to 1% of lung transplant patients [1]. Another large series published after this review reported a prevalence of 4/462 (0.9%) [5]. The reported cases include early and advanced NSCLC, as well as small cell lung cancer, treated with various modalities including radiotherapy, chemotherapy, surgery and best supportive care. While SABR was not employed for lung cancer arising from donor lung within any of these large reports, a literature search performed by our research team identified one other case of early-stage NSCLC arising from donor lung treated with SABR [6]. This patient previously received a single-lung transplant and SABR treatment was similarly well-tolerated, with the patient remaining disease-free after 22 months of follow-up. To our knowledge, our patient represents the first reported case where SABR was used to treat an early-stage NSCLC arising from donor lung following double-lung transplantation.

Guidelines for local treatment of early-stage NSCLC likely still apply for cancer arising from donor lung [7, 8]. Surgery remains the mainstay of treatment in medically-operable Stage I/II NSCLC, while radiotherapy is indicated with radical intent for medically-inoperable early-stage disease and Stage III NSCLC. However, data is lacking on the interaction between immunosuppression and oncologic treatments. Traditional chemotherapy seems to be tolerated after transplantation [9], though there is a paucity of information regarding newer systemic therapy agents such as tyrosine kinase inhibitors and immunotherapeutic agents. The importance of an intact immune system in facilitating optimal radiation therapy response is being actively researched [10], though the precise implications of an immunosuppressed state during radiation therapy is not clearly understood.

The lung cancer described in the present case arose in lungs from an extended-criteria lung donor [11, 12]. These donors have a preponderance of being older, with some baseline impairment of pulmonary function and/or additional smoking history. Accepting potential donors with these less favorable characteristics allow for improved access to lung transplantation, and have become gradually more accepted within transplant centers over the past two decades [13]. Inclusion of extended-criteria lung donors appears to be associated with an increase in short-term adverse outcomes, although there does not appear to be a major impact on long-term outcomes compared to standard-criteria donors [14]. It is unclear whether extended-criteria donor lungs are associated with any increased risk of malignancy. Regardless, the overall low prevalence of primary lung cancer arising from donor lungs, despite immunosuppression, emphasizes the rarity of such a scenario. Such rarity, however, also makes prevention through screening difficult, due to the high number of expected false positives and low overall cost-effectiveness. Until more data is available, the benefits of expanding access to lung transplantation likely outweighs the relatively small risk of malignancy when using extended-criteria donor lungs.

The present case also highlights the difficulties faced by clinicians during follow-up for lung radiotherapy. In the subacute timeframe, it can be challenging to distinguish respiratory events due to co-existing pulmonary comorbidities from radiation pneumonitis. Differential etiologies such as viral/bacterial pneumonia, chronic obstructive pulmonary disease exacerbation, and interstitial lung disease exacerbation should be addressed concurrently based on clinical features. In the long term, identifying recurrences where the perilesional lung becomes fibrotic is a unique challenge post-SABR. Any potentially adverse features [2] should be interpreted through multidisciplinary collaboration between thoracic radiation oncologists and radiologists. Of note, the development of computerized algorithms (radiomics) to identify subtler radiographic features in this setting is an active area of research [15].

## Conclusion

We demonstrate the feasibility and safety of full-dose SABR for an early-stage NSCLC arising from donor lung in a double-lung transplant recipient. A larger repertoire of patients with adequate follow-up is required to determine SABR’s oncologic effectiveness in this population of chronically immunosuppressed patients.

## Abbreviations

CLAD: Chronic lung allograft dysfunction
CT: Computed tomography
FEV1: Forced expiratory volume in 1 s
FVC: Forced vital capacity
MR: Magnetic resonance
NSCLC: Non-small cell lung cancer
PET-CT: Positron emission tomography-computed tomography
PTLD: Post-transplant lymphoproliferative disorder
SABR: Stereotactic ablative radiotherapy
V20: Volume receiving at least 20 Gy
